# 204. Mucosal Cytokine Profiles in Children with COVID-19

**DOI:** 10.1093/ofid/ofab466.000

**Published:** 2021-12-04

**Authors:** Shira H Cohen, Cameron Mertz, Rebecca M Glowinski, Sara Mertz, Fang Ye, Zhaohui Xu, Lauren Miller, Colin L Peachey, Amber Wolfe, Traci Pifer, Kathy Everhart, Amy Leber, Pablo J Sanchez, Octavio Ramilo, Asuncion Mejias

**Affiliations:** 1 Nationwide Children’s Hospital, Columbus, Ohio; 2 N/A columbus, Ohio; 3 Nationwide Children’s Hospital - The Ohio State University, Columbus, Ohio

## Abstract

**Background:**

The mechanisms associated with COVID-19 in children are not well understood. We sought to define the differences in nasopharyngeal (NP) cytokine profiles according to clinical presentation in children with COVID-19.

**Methods:**

Single-center, prospective study in 137 children and adolescents < 21 years of age hospitalized with COVID-19, and 35 age, sex and race matched pre-pandemic (2016-2019) healthy controls. Children with COVID-19 were categorized according to their clinical presentation in: COVID-19-symptomatic; COVID-19-screening, and multisystem inflammatory syndrome (MIS-C). NP swabs were obtained within 24 hours of admission to measure SARS-CoV-2 loads by rt-PCR, and a 92-cytokine panel. Unsupervised and supervised analysis adjusted for multiple comparisons were performed.

**Results:**

From 3/2020 to 1/2021, we enrolled 76 COVID-19-symptomatic children (3.5 [0.2-15.75] years); 45 COVID-19-screening (11.1 [4.2-16.1] years), and 16 MIS-C (11.2 [5.9-14.6] years). Median NP SARS-CoV-2 loads were higher in COVID-19-symptomatic *versus* screening and MIS-C (6.8 vs 3.5 vs 2.82 log_10_ copies/mL; p< 0.001). Statistical group comparisons identified 15 cytokines that consistently differed between groups and were clustered in three functional categories: (1) antiviral/regulatory, (2) pro-inflammatory/chemotactic, and (3) a combination of (1) and (2); (**Fig 1**). All 15 cytokines were higher in COVID-19-symptomatic *versus* controls (p< 0.05). Similarly, and except for TNF, CCL3, CCL4 and CCL23, which were comparable in COVID-19-symptomatic and screening patients, the remaining cytokines were higher in symptomatic children (p< 0.05). PDL-1 (p=0.01) and CCL3 (p=0.03) were the only cytokines significantly decreased in children with MIS-C *versus* symptomatic COVID-19 children.

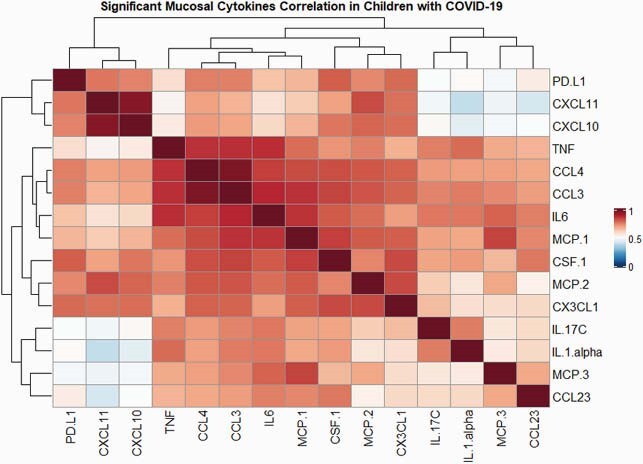

The 15 cytokines identified by multiple comparisons were correlated using Person’s in R software. Red reflects a positive correlation and blue a negative correlation with the intensity of the color indicating the strength of the association.

**Conclusion:**

Children with symptomatic COVID-19 demonstrated higher viral loads and greater mucosal cytokines concentrations than those identified via screening, whereas in MIS-C concentrations of regulatory cytokines were decreased. Simultaneous evaluation of viral loads and mucosal immune responses using non-invasive sampling could aid with the stratification of children and adolescents with COVID-19 in the clinical setting.

**Disclosures:**

Octavio Ramilo, MD, Adagio (Consultant)Bill & Melinda Gates Foundation (Grant/Research Support)Janssen (Grant/Research Support)Lilly (Consultant)Merck (Consultant, Grant/Research Support)NIH (Grant/Research Support)Pfizer (Consultant)SANOFI (Board Member) Asuncion Mejias, MD, PhD, MsCS, Janssen (Grant/Research Support, Advisor or Review Panel member)Merck (Grant/Research Support, Advisor or Review Panel member)Roche (Advisor or Review Panel member)Sanofi (Advisor or Review Panel member).

